# Bayesian Inference of Forces Causing Cytoplasmic Streaming in *Caenorhabditis elegans* Embryos and Mouse Oocytes

**DOI:** 10.1371/journal.pone.0159917

**Published:** 2016-07-29

**Authors:** Ritsuya Niwayama, Hiromichi Nagao, Tomoya S. Kitajima, Lars Hufnagel, Kyosuke Shinohara, Tomoyuki Higuchi, Takuji Ishikawa, Akatsuki Kimura

**Affiliations:** 1 Department of Genetics, School of Life Science, SOKENDAI (The Graduate University for Advanced Studies), Mishima, Japan; 2 Cell Architecture Laboratory, Structural Biology Center, National Institute of Genetics, Mishima, Japan; 3 Transdisciplinary Research Integration Center, Research Organization of Information and Systems, Tokyo, Japan; 4 Research and Development Center for Data Assimilation, The Institute of Statistical Mathematics, Tachikawa, Japan; 5 Laboratory for Chromosome Segregation, RIKEN Center for Developmental Biology (CDB), Kobe, Japan; 6 Cell Biology and Biophysics Unit, EMBL Heidelberg, Heidelberg, Germany; 7 Developmental Genetics Group, Graduate School of Frontier Biosciences, Osaka University, Suita, Osaka, Japan; 8 Biological Flow Studies Laboratory, Department of Bioengineering and Robotics, Tohoku University, Sendai, Japan; Bioinformatics Institute, SINGAPORE

## Abstract

Cellular structures are hydrodynamically interconnected, such that force generation in one location can move distal structures. One example of this phenomenon is cytoplasmic streaming, whereby active forces at the cell cortex induce streaming of the entire cytoplasm. However, it is not known how the spatial distribution and magnitude of these forces move distant objects within the cell. To address this issue, we developed a computational method that used cytoplasm hydrodynamics to infer the spatial distribution of shear stress at the cell cortex induced by active force generators from experimentally obtained flow field of cytoplasmic streaming. By applying this method, we determined the shear-stress distribution that quantitatively reproduces in vivo flow fields in *Caenorhabditis elegans* embryos and mouse oocytes during meiosis II. Shear stress in mouse oocytes were predicted to localize to a narrower cortical region than that with a high cortical flow velocity and corresponded with the localization of the cortical actin cap. The predicted patterns of pressure gradient in both species were consistent with species-specific cytoplasmic streaming functions. The shear-stress distribution inferred by our method can contribute to the characterization of active force generation driving biological streaming.

## Introduction

Cellular components require proper positioning to perform their functions within the cell. The generation of active forces is essential for moving intracellular materials to their target locations; motor proteins and cytoskeletons are the force generators responsible this transport [1]. Clarifying the distribution of active forces—i.e., where and to what degree these forces are generated—is critical for understanding the mechanisms of intracellular transport. In cases where transported components are directly tethered to the force generators, it can be assumed that the drag force is proportional to the velocity, according to Stokes’ law. However, inferring force is difficult when active force generation at one location moves cellular components at a distal site within the cell via indirect interactions controlled by the hydrodynamic properties of the cytoplasm [2]. Cell-wide cytoplasmic movement, cytoplasmic streaming, is an example of such movement. Cytoplasmic streaming is described in several types of animal and plant cells as hydrodynamic motion driven by active force generators at the cell cortex [3–10]. Specifically, these generators undergo oriented movement at the cell cortex, inducing shear stress that drives movement of the entire cytoplasm. The shear-stress distribution should directly reflect the position and magnitude of active force generation, but its characterization is challenging.

**Fig 1:**
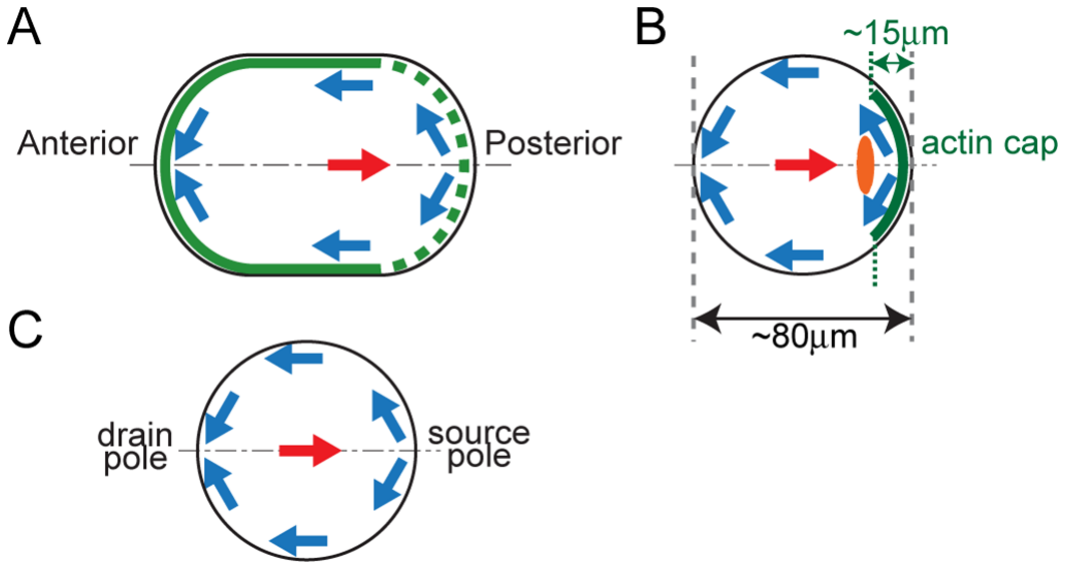
Schema depicting flow fields of cytoplasmic streaming. Cell boundaries are shown in black, and flow directions near the cell cortex and at the cell center are shown in blue and red arrows, respectively. (A) Cytoplasmic streaming in the *C*. *elegans* embryo. The myosin-II-enriched region is shown in green. (B) Cytoplasmic streaming in the mouse oocyte. The Arp2/3-enriched actin cap is shown in green, and the meiotic spindle is shown in orange. (C) Definitions for source and drain poles used in this study.

In the *Caenorhabditis elegans* embryo, cytoplasmic streaming is observed at the one-cell stage and contributes to the establishment of embryo polarity [11,12] (Fig 1A, S1 Movie). The active force generator for this flow is the network of actin filaments and non-muscle myosin II (NMY-2). The network is concentrated at the cell cortex, and contracts to produce movement in a posterior-to-anterior direction [5]. Based on measurements of cortical tension, it has been proposed that contraction in the anterior region drives long-range flow, since internal viscosity overrides external friction [10]. When the cortical myosin moves anteriorly, materials in the central cytoplasm move in the opposite direction (i.e., posteriorly) [11]. In a previous study, we speculated that anteriorly directed shear stress generation at the cell cortex drives hydrodynamic flow in the opposite direction within the cytoplasm, and tested this hypothesis by reproducing the velocity field of the entire cytoplasm using a computer simulation of hydrodynamic forces [8]. The fact that not only cytoplasmic granules but also injected micro-beads are carried by cytoplasmic flow supports its hydrodynamic nature [13].

Cytoplasmic streaming with similar bi-directional flow field is also observed in mouse oocytes during meiosis II [7] (Fig 1B, S2 Movie). The flow along the cell cortex and in the central cytoplasm is directed away from the actin-rich cortical cap and towards the cap, respectively. This streaming is thought to facilitate the extrusion of the second polar body by pushing the meiotic spindle towards the cap [7]. Based on the similarity between this flow field and that observed in *C*. *elegans* embryos, we speculated that cytoplasmic flow in mouse oocytes is driven by the cortical flow coupled with the hydrodynamic properties of the cytoplasm. However, the actin cap covers only a limited region of the cortex, and it is therefore unclear whether the shear stress within this region is sufficient to move an extensive region of the cytoplasm.

In this study, we present a method to computationally infer the spatial distribution of shear stress at the cell cortex driving cytoplasmic streaming. Our inference is based on fitting the cytoplasmic flow field calculated with the hydrodynamic simulation to experimentally obtained values using data assimilation (DA), a Bayesian inference technique used for integrating hypothetical and empirical derived data [14–16]. We applied our method to determine the distribution of shear stress in *C*. *elegans* embryo and mouse oocyte. The resulting estimates allowed us to test whether the active force generation occurs exclusively within the actin cap of the mouse oocyte. In addition, we compared the characteristics of the shear stress driving cytoplasmic streaming in *C*. *elegans* embryos and mouse oocytes, from which we speculate on how active force generation is spatially organized so as to fulfill distinct functions in the two species. The shear-stress distribution obtained by the DA-based method can provide insight into cytoplasmic streaming processes in many species.

## Materials and Methods

### Assumptions for modeling cytoplasmic streaming in *C*. *elegans* embryos and mouse oocytes

Our hydrodynamic model of cytoplasmic streaming was based on three assumptions. We first assumed that cytoplasmic flow can be described by Stokes’ equation—i.e., the inertia in the system can be neglected and the cytoplasm is a Newtonian fluid for which elasticity can also be discounted. The Reynolds’ numbers for cytoplasmic streaming in *C*. *elegans* and mouse are in the orders of 10^−12^ and 10^−13^, respectively [17]; these small values indicate that inertia in these systems is negligible. It has also been shown that elasticity in *C*. *elegans* cytoplasmic streaming is also negligible [10], and that the cytoplasm of *Drosophila* oocytes can be considered as a Newtonian fluid for the purposes of cytoplasmic streaming [18].

Our second assumption was that shear stress generated at the cortex is the main force driving cytoplasmic streaming and that a negligible amount of force is generated within the cytoplasm. In *C*. *elegans* embryos and mouse oocytes, directed motion of the cortical actin network at the cell surface is presumed to generate shear stress [5,7,10]. The molecules generating the force required for cytoplasmic streaming such as actin, myosin II, and actin-related protein (Arp)2/3 are localized almost exclusively at and move along the cell cortex during this process [3,5,7,11]. The velocity fields of cytoplasmic streaming in both species were assumed to be consistent with that of the hydrodynamic simulation in which the cytoplasm was considered as a Newtonian fluid and cortical shear stress as the sole active force [7,8].

The third assumption for our model was that cytoplasmic streaming is symmetric about the anterior-posterior (AP) axis in *C*. *elegans* embryos, and about the axis passing through the center of the cell and the meiotic spindle in mouse oocytes. We refer to the two poles of the cell on this axis of symmetry as source and drain poles (Fig 1C); these represent the points from which shear stress diverges, and into which shear stress converges, respectively. Axial symmetry around the source-drain axis has been previously demonstrated in the context of cytoplasmic streaming in mouse oocytes [7]; we demonstrate the same for *C*. *elegans* embryos in the present study.

### 3-Dimensional (3D) visualization of cytoplasmic streaming in *C*. *elegans* embryos

To visualize cytoplasmic streaming in *C*. *elegans* embryos, we used the RT130 [vitellogenin (VIT)-2::GFP] strain (*Caenorhabditis* Genetics Center, University of Minnesota College of Biological Sciences, Saint Paul, MN, USA) in which yolk granules uniformly distributed in the cytoplasm are fluorescently labeled. The multi-view selective plane illumination microscopy (MuVi-SPIM) approach [19] was used to record cytoplasmic streaming patterns for extend periods of time with a microscope harboring two illumination (10×, NA = 0.3) and two detection (25×, NA = 1.1) objectives that enabled fast and light-efficient acquisition of 3D datasets (Nikon, Tokyo, Japan). Adult worms were anesthetized by embedding in agar cylinders placed in a levamisole-M9 solution. 3D fluorescence datasets (41–81 planes with 1-μm spacing) of embryos inside the adult worms were acquired from four views (two opposing cameras with 0° and 90° sample rotation). This cycle was repeated every 5–8 s depending on the experiment. The highest-quality images obtained from the four views were processed using the 3D particle image velocimetry (PIV) algorithm.

### 3D PIV

We developed a 3D PIV algorithm by extending the previously described 2D PIV algorithm [20] into 3D (S1 Code). Our algorithm first quantified the pixel-level motion of the cytoplasm. Specifically, from the brightness *I*(*x*, *y*, *z*, *t*) of the position (*x*, *y*, *z*) at time *t*, we estimated the pixel-level motion (*ξ*, *η*, *λ*) at position (*x*, *y*, *z*) during the period (*t* to *t* + *Δt*) that maximized the function *R*(*ξ*, *η*, *λ*) = Σ_*A*_ *I*(*x*, *y*, *z*, *t*)*I*(*x* + *ξ*,*y* + *η*,*z* + *λ*,*t* + Δ*t*). The summation passed through the 3D interrogation window *A*. To reduce erroneous estimation, (*ξ*, *η*, *λ*) was iteratively estimated three times with successively smaller 3D interrogation window sizes. The first, second, and third interrogation window sizes were (33 × 33 × 1), (29 × 29 × 1), and (25 × 25 × 1), and the values for (*ξ*, *η*, *λ*) that would maximize *R*(*ξ*, *η*, *λ*) were searched within the range of (±5, ±5, 0), (±2, ±2, ±1), and (±1, ±1, 0), respectively. These ranges were kept small in the *z* direction since pixel size in this direction (1.0 μm/pixel) was roughly four times greater than those in the *x* and *y* directions (0.26 μm/pixel).

After quantifying the pixel-level displacement, 3D sub-pixel displacements (*u*, *v*, *w*) were calculated using a supercomputer with the following matrix equation:

$$\left(\begin{array}{ccc} \sum_{A}f_{x}^{2} & \sum_{A}f_{x}f_{y} & \sum_{A}f_{x}f_{z}\\ \sum_{A}f_{x}f_{y} & \sum_{A}f_{y}^{2} & \sum_{A}f_{y}f_{z}\\ \sum_{A}f_{x}f_{z} & \sum_{A}f_{y}f_{z} & \sum_{A}f_{z}^{2} \end{array}\right)\left(\begin{array}{c} u\\ v\\ w \end{array}\right)=\left(\begin{array}{c} \sum_{A}f_{x}f_{t}\\ \sum_{A}f_{y}f_{t}\\ \sum_{A}f_{z}f_{t} \end{array}\right)$$ (1)

Displacement in pixel units was calculated by adding (*u*, *v*, *w*) to (*ξ*, *η*, *λ*), after which pixel-unit displacement was converted into units of μm/s using pixel (voxel) size (0.26 μm/pixel in the *x* and *y* directions and 1.0 μm/pixel in the *z* direction) and imaging interval. The coordinates (*x*, *y*, *z*) of the anterior and posterior poles of the cells in the images were identified using Imaris software (Bitplane, Zurich, Switzerland). Velocity components along the AP axis were calculated using MATLAB software (MathWorks, Natick, MA, USA).

### 2D visualization and PIV of cytoplasmic streaming in *C*. *elegans* embryos and mouse oocytes

We collected germinal vesicle-stage oocytes from the ovaries of female BDF1 mice sacrificed by cervical dislocation. This study was carried out in accordance with the guidelines for animal experimentation of RIKEN Center for Developmental Biology (CDB), Kobe, Japan. The protocol was approved by the Animal Care and Use Committee of RIKEN CDB (approval #AH23-05-04). Female BDF1 mice were purchased from SLC Japan (Shizuoka, Japan). Oocytes were cultured in M2 medium at 37°C for 12–15 h to allow meiotic maturation. Cytoplasmic streaming was recorded in mature oocytes at metaphase of meiosis II. Transmitted-light images of oocytes were recorded at 1-min intervals using an LSM710 microscope (Zeiss, Jena, Germany) equipped with a C-Apochromat 40×/1.2W Corr M27 objective lens and a XLmultiS1 incubator (PeCon, Erbach Germany) set at 37°C. Cytoplasmic flow was quantified by 2D PIV as described [8] (S3 Code). The flow field was almost stationary at this stage, and we used the average flow field over 50 min as the value for each oocyte. For *C*. *elegans*, we used quantitative data obtained in our previous study [8], which includes velocity fields from when the flow was fastest in individual embryos. The PIV results that were used in DA were not identical to those of the previous study although the raw data were identical, since the data points were relocated (see *boundary shapes used in DA*).

### Fluid dynamics simulation performed using the streaming function-vorticity method

Because we assumed that the cytoplasm was a Newtonian fluid, the Navier-Stokes equation—which applies to viscous fluids—was used to describe the hydrodynamics of the cytoplasm. This equation can be solved using the stream function-vorticity method for 2D incompressible flow [21], which can also be applied to 3D flow with axial symmetry. The stream function (*Ψ*) and vorticity (*Ω*) are introduced to fulfill $v_{z}=\frac{1}{r}\frac{\partial \Psi }{\partial r}$, $v_{r}=-\frac{1}{r}\frac{\partial \Psi }{\partial z}$, and $\Omega =\frac{\partial v_{r}}{\partial z}-\frac{\partial v_{z}}{\partial r}$. Here, *z* and *r* refer to the coordinate along the source-drain axis and in the radial direction, respectively, and *v*_*z*_ and *v*_*r*_ refer to flow velocity along directions *z* and *r*, respectively. Under conditions of low Reynolds number, the steady state flow follows the following equations and boundary conditions: the vorticity (*Ω*)-transport equation for Stokes flow:

$$\frac{\partial^{2}\Omega }{\partial z^{2}}+\frac{\partial^{2}\Omega }{\partial r^{2}}+\frac{1}{r}\frac{\partial \Omega }{\partial r}-\frac{\Omega }{r^{2}}=0$$ (2)

the Poisson equation for the stream function *Ψ*:

$$\frac{\partial^{2}\Psi }{\partial z^{2}}+\frac{\partial^{2}\Psi }{\partial r^{2}}-\frac{1}{r}\frac{\partial \Psi }{\partial r}+r\Omega =0$$ (3)

and the boundary conditions:

Ψ = Ω = 0 on the central axis; and

Ψ = 0, $\Omega =-\frac{\tau }{\mu }+\frac{2v_{t}}{R}$ at the cell surface.

In this boundary condition, *τ*, *μ*, *v*_*t*_, and *R* refer to wall shear stress, viscosity, flow velocity along the surface, and the curvature radius at individual positions on the cell surface, respectively. We specified the shear-stress distribution in our calculations using the B-spline function. All variables and parameters were non-dimensionalized, and the viscosity and short radius of the cell were set to 1.0.

We introduced a boundary-fitted curvilinear coordinate grid [22] to solve the above equations. For *C*. *elegans*, the source-drain axis and rotational radius were divided into 56 and 14 grids, respectively (corresponding to 55 and 13 μm, respectively, in the actual embryo). For mouse, the source-drain axis and rotational radius were divided into 25 and 13 grids, respectively. The original (*z*, *r*) coordinate system was transformed to a (*z*, *s*) coordinate system, where s is defined by *r*/*F*(*z*) and *F*(*z*) is the radius of the cell at position *z*. Using *s* and *z*, Eqs 2 and 3 can be re-written as:

$$\left(S_{r}^{2}+S_{z}^{2}\right)\frac{\partial^{2}\Omega }{\partial s^{2}}+2S_{z}\frac{\partial^{2}\Omega }{\partial s\partial z}+\frac{\partial^{2}\Omega }{\partial z^{2}}+\left(S_{zz}+S_{rr}+\frac{S_{r}}{F(z)s}\right)\frac{\partial \Omega }{\partial s}-\frac{\Omega }{{\left(F(z)s\right)}^{2}}=0$$ (4)

$$\left(S_{r}^{2}+S_{z}^{2}\right)\frac{\partial^{2}\Psi }{\partial s^{2}}+2S_{z}\frac{\partial^{2}\Psi }{\partial s\partial z}+\frac{\partial^{2}\Psi }{\partial z^{2}}+\left(S_{zz}+S_{rr}-\frac{S_{r}}{F(z)s}\right)\frac{\partial \Psi }{\partial s}+F(z)s\Omega =0,$$ (5)

where

$S_{r}=\frac{1}{F(z)}$, $S_{z}=-\frac{s}{F(z)}\frac{dF(z)}{dz}$, $S_{zz}=\frac{s}{F(z)}\left\{\frac{2}{F(z)}{\left(\frac{dF(z)}{dz}\right)}^{2}-\frac{d^{2}F(z)}{dz^{2}}\right\}$, and *S*_*rr*_ = 0. The partial of (*z*, *s*) was connected with that in the (*z*, *r*) system using the equation:

$$\left(\begin{array}{c} \frac{\partial }{\partial r}\\ \frac{\partial }{\partial z} \end{array}\right)=\left(\begin{array}{cc} S_{r} & 0\\ S_{z} & 1 \end{array}\right)\left(\begin{array}{c} \frac{\partial }{\partial s}\\ \frac{\partial }{\partial z} \end{array}\right)$$ (6)

The vorticity-transport equation (Eq 4) was solved using the Euler explicit method, and the Poisson equation (Eq 5) was solved using the successive over-relaxation method [22] with the above-mentioned boundary conditions.

### DA-based estimation of shear stress

**Fig 2:**
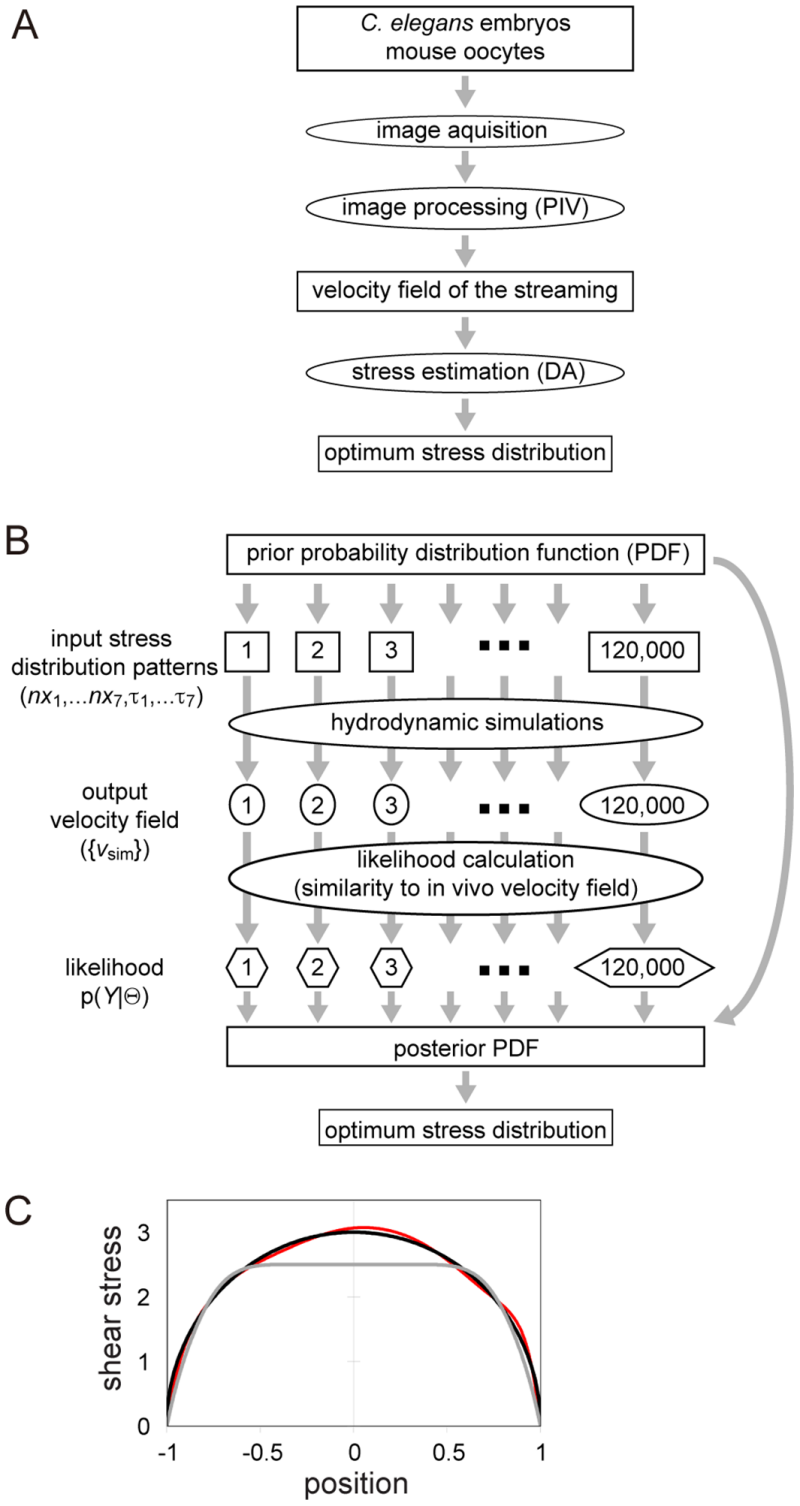
The DA method and a benchmark test. (A) Overall scheme for estimating the optimum distribution of stress. (B) Scheme used to automatically calculate optimum stress distribution using the DA method developed in this study. We used *N*_*Sample*_ = ~120,000 patterns of the shear-stress distribution. (C) In the benchmark test, we tested if we could estimate the shear-stress distribution *τ*(*z*) = 3 × (1 − *z*^2^)^0.5^ driving streaming in a sphere (black line). Estimation procedures were applied starting from a prior stress distribution (gray line). The result of the estimation is shown in red. The results converged to the correct answer, which is indicated by the black line. Dimensionless units were used for both the position and shear stress.

The DA procedure is used to estimate the state of a system by statistically comparing experimental and simulation data based on Bayesian statistics (Fig 2A). In this study, DA was used to obtain the posterior probability density function (PDF) p(***Θ***|***Y***), which refers to the probability of the model parameter set ***Θ*** contingent on experimental data ***Y***. Specifically, ***Θ*** contains the positions of the nodes of a B-spline function, which describes the shear-stress distribution (see *Using B-spline to specify shear-stress distribution* below), and ***Y*** denotes the velocity field experimentally determined by the PIV method. Bayes’ theorem, $p(\Theta |Y)=\frac{p(Y|\Theta )p(\Theta )}{\int p(Y|\Theta )p(\Theta )d\Theta }$, indicates that the posterior PDF, p(***Θ***|***Y***), can be obtained as a product of the prior PDF, p(***Θ***), and the likelihood, p(***Y***|***Θ***), since the denominator—which represents the normalization factor—is constant. The prior PDF, p(***Θ***), is an a priori assumption made based on knowledge of the parameters (S1 Fig). The likelihood, p(***Y***|***Θ***), is the plausibility of ***Y*** conditioned by the given ***Θ***.

To obtain the posterior PDF, we used the following procedure (Fig 2B and S3 Code). We first sampled a sufficient number of ***Θ***_*i*_ (*i* = 1 to *N*_*Sample*_) from the prior probability p(***Θ***). In this study, we set *N*_*Sample*_ as about 120,000. We then performed a hydrodynamic simulation for each ***Θ***_*i*_, and calculated the likelihood p(***Y***|***Θ***_*i*_) (*i* = 1 to *N*_*Sample*_) by comparing the simulation results with the experimental data. Since we carried out sampling based on the prior PDF, the (non-normalized) posterior probability for *i*-th sample was identical to the likelihood for that sample. Finally, we estimated the optimum parameter set by $\frac{\sum_{i=1}^{M}\Theta_{i}p\left(\Theta_{i}|Y\right)}{\sum_{i=1}^{M}p\left(\Theta_{i}|Y\right)}$, where the summation was applied to *M* samples featuring the largest posterior values. In this study, *M* was set as 100. We performed two rounds of estimations: first, we estimated ***Θ***, and then performed the second round of estimation using the estimated ***Θ*** as the average of Gaussian distribution in the prior.

### Using B-spline to specify shear stress distribution

Shear-stress distribution was parametrically specified using the B-spline function *τ*(*z*), where *z* is the position along the source-drain axis and *τ*(*z*) is the amplitude of shear stress at position *z* (S1 Fig). The B-spline function interpolates a given set of nodes featuring the *z*-*r* coordinates of (*nz*_*i*_, *τ*_*i*_) (*i* = −2 to *N*_*nodes*_ + 3). In our analysis, *N*_*nodes*_ = 7. To make *τ*(*z*) = 0 at the source and drain poles, we set (*nz*_*i*_, *τ*_*i*_) = (−*R*, 0) for *i* = −2, −1, and 0, and (*nz*_*i*_, *τ*_*i*_) = (R, 0) for *i* = *N*_*nodes*_ + 1, *N*_*nodes*_ + 2, and *N*_*nodes*_ + 3, where *R* is half the distance between the source and drain poles. The *nz*_*i*_ for *i* = 1 to *N*_*nodes*_ was set as shown in (S1A and S1B Fig); *τ*_*i*_ for *i* = 1 to *N*_*nodes*_ corresponded to shear-stress amplitude at the individual positions *nz*_*i*_, and was the component of our model parameter ***Θ***; namely, ***Θ*** = (*θ*_*1*_, *θ*_*2*_, …, *θ*_*Nnodes*_) = (*τ*_*1*_, *τ*_*2*_, …, *τ*_*Nnodes*_).

### Setting prior values

The prior p(***Θ***) values were set as the product of each ***θ***_*i*_ {*i* = 1 to *N*_*nodes*_}, which were assumed to follow an independent Gaussian distribution P_*i*_(***θ***_***i***_) {i = 1 to *N*_*nodes*_}. This can be written as

$$p(\Theta )=\prod_{i=1}^{N_{nodes}}P_{i}\left(\theta_{i}\right)$$ (7)

Examples of the average and standard deviation calculated for each Gaussian distribution used for *C*. *elegans* embryos and mouse oocytes are shown in S1A and S1B Fig, respectively. In the case of *C*. *elegans*, the average of P_*i*_(***θ***_*i*_) was set by manually fitting the B-spline to the cortical velocity of individual embryos. In the case of mouse oocytes, we did not use distinct priors for individual oocytes; instead we used an identical prior (S1B Fig). The average of *P*_*i*_(***θ***_*i*_) was set as 2.5 in the region near the source pole, whereas in other locations of the non-dimensionalized system, the value was set as 0.5. We set the standard deviation of P_*i*_(***θ***_*i*_) as 0.75 in our estimation in the non-dimensionalized system. In addition, we sampled shear-stress distribution under the restriction that all sampled ***θ***_*i*_ were > 0. This restriction was set under the assumption that the shear stress was acting in the direction from the source to the drain pole. The initial non-dimensional unit of stress was converted into a realistic unit based on the length and time scales of cytoplasmic streaming in individual cells.

### Setting likelihood values

The likelihood p(*Y*|***Θ***) was calculated by comparing the simulation result obtained using the specific parameter set ***Θ*** with experimental data *Y* at a total of *N*_*location*_ points (S1 Dataset). We set the *z*- and *r*-axes such that they were parallel and orthogonal, respectively, to the source-drain axis. By allowing *v*^*i*^_*z_sim*_ and *v*^*i*^_*r_sim*_ to be the *z* and *r* components of the simulated velocity at the *i*-th observation point, and *v*^*i*^_*z_exp*_ and *v*^*i*^_*r_exp*_ to be the experimental data at the point, we defined the likelihood as

$$p\left(Y|\Theta \right)=\prod_{i=1}^{N_{location}}P_{Gauss}\left(v_{z\_sim}^{i},v_{r\_sim}^{i},v_{z\_exp}^{i},v_{r\_exp}^{i},\sigma_{z}^{2},\sigma_{r}^{2},\sigma_{zr}\right)$$ (8)

where $P_{Gauss}\left(v_{z\_sim}^{i},v_{r\_sim}^{i},v_{z\_exp}^{i},v_{r\_exp}^{i},\sigma_{z}^{2},\sigma_{r}^{2},\sigma_{zr}\right)$ is the 2D Gaussian distribution for (*v*_*z*_, *v*_*r*_) with a mean vector (*v*^*i*^_*z_exp*_, *v*^*i*^_*r_exp*_) and a covariance matrix $\left(\begin{array}{cc} \sigma_{z}^{2} & \sigma_{zr}\\ \sigma_{zr} & \sigma_{r}^{2} \end{array}\right)$, in which *σ*_*z*_^2^, *σ*_*r*_^2^, and *σ*_*zr*_ are assumed to be independent of *i*. *Y* is the 2*N*_*location*_ dimensional vector {*v*^*i*^_*z_exp*_, *v*^*i*^_*r_exp*_}(*i* = 1,…, *N*_*location*_). *σ*_*z*_^2^, *σ*_*r*_^2^, and *σ*_*zr*_ that maximize the likelihood was calculated by $\sigma_{z}^{2}=\frac{1}{N_{location}}\left\{\sum_{i=1}^{N_{location}}{\left(v_{z\_sim}^{i}-v_{z\_exp}^{i}\right)}^{2}\right\}$; $\sigma_{r}^{2}=\frac{1}{N_{location}}\left\{\sum_{i=1}^{N_{location}}{\left(v_{r\_sim}^{i}-v_{r\_exp}^{i}\right)}^{2}\right\}$; and $\sigma_{zr}=\frac{1}{N_{location}}\left\{\sum_{i=1}^{N_{location}}\left(v_{z\_sim}^{i}-v_{z\_exp}^{i}\right)\left(v_{r\_sim}^{i}-v_{r\_exp}^{i}\right)\right\}$. By substituting these values into Eq 8, we obtained p(*Y*|***Θ***) for individually sampled ***Θ***.

### Boundary shapes used in DA

The shape of the *C*. *elegans* embryo was modeled as capsule-shape—i.e., a cylinder with hemispheres of radius 1.0 at both ends. The long axis length was 55/13 based on the aspect ratio of real cells (~55 and ~26 μm for the long and short axes, respectively). The shape of the mouse oocyte was approximated as a sphere with a radius of 1.0.

The positions inside the cell measured in experiments were repositioned in order to fit within the aforementioned boundary used in the simulation. In the case of the *C*. *elegans* embryo, we obtained images of VIT-2-GFP expressed in individual embryos that were then rotated using ImageJ (National Institutes of Health, Bethesda, MD, USA) so that the horizontal (*z*) axis was aligned with the AP (source-drain) axis [8]. Images were binarized using ImageJ in order to distinguish the inside from the outside of the cells, after which the points that featured the maximum (*Z*_*max*_) and minimum (*Z*_*min*_) values of the horizontal coordinate inside individual embryos were designated as the source and drain poles, respectively, with the distance between them normalized to 55/13. Next, the distances of individual PIV data points from the source pole in the horizontal direction were set as $\frac{55}{13}\frac{\left(Z_{max}-Z_{PIV}\right)}{\left(Z_{max}-Z_{min}\right)}$ in the non-dimensionalized system, where *Z*_*PIV*_ is the *z* coordinate of individual PIV data points. To determine a value for *r*, we first calculated the radius of the cell boundary used in the simulation at individual *z* coordinates. Lastly, the locations of PIV data points, which featured minimum and maximum values of *r* for individual *z* values, were fitted so that they were located at the lower and upper radius positions in the simulation cell boundary, respectively; in addition, the locations of the data points between the upper and lower radii were interpolated linearly at individual *z* coordinates.

In the case of mouse oocytes, we rotated the image of the cell such that the drain and the source poles were to the left and right, respectively. We obtained the upper and lower outlines of cells in the images using ImageJ. With this outline, we defined *Z*_*max*_ and *Z*_*min*_, after which the *z* distance of PIV data points from the source pole were set as $2.0\times \frac{\left(Z_{max}-Z_{PIV}\right)}{\left(Z_{max}-Z_{min}\right)}$. The subsequent steps were the same as those used for *C*. *elegans* embryos. The velocity component along the cell cortex was calculated as the inner product of the velocity vector at the cortex and the unit vector along the cell boundary.

### Calculation of pressure gradient

To compare the pressure gradients resulting from shear stress distribution in *C*. *elegans* and mouse, we calculated pressure gradients in identical shapes (spherical or capsule). We first calculated flow velocity field with the estimated shear stress value. Total shear stress was normalized to 1.5 in our non-dimensionalized system.

We next calculated the pressure using the calculated flow velocity field by integrating the Stokes equation, which can be written as follows using (*z*, *r*) as variables:

$$\frac{\partial p}{\partial z}=\frac{1}{r}\frac{\partial u_{z}}{\partial r}+\frac{\partial^{2}u_{z}}{\partial r^{2}}+\frac{\partial^{2}u_{z}}{\partial z^{2}}$$ (9)

$$\frac{\partial p}{\partial r}=\frac{1}{r}\frac{\partial u_{r}}{\partial r}+\frac{\partial^{2}u_{r}}{\partial r^{2}}+\frac{\partial^{2}u_{r}}{\partial z^{2}}$$ (10)

where *p* and *u*_*z*_ represent the pressure and velocity, respectively, in the *z* direction. Using (*z*, *r*) as variables, Eqs 9 and 10 were converted to equations using (*z*, *s*) with Eq 6, and were discretized using the finite-difference method. A first-order finite-difference scheme was used for all grid points.

## Results

### 3D visualization and velocity quantification indicate high axial symmetry of the flow field in the *C*. *elegans* embryo

**Fig 3:**
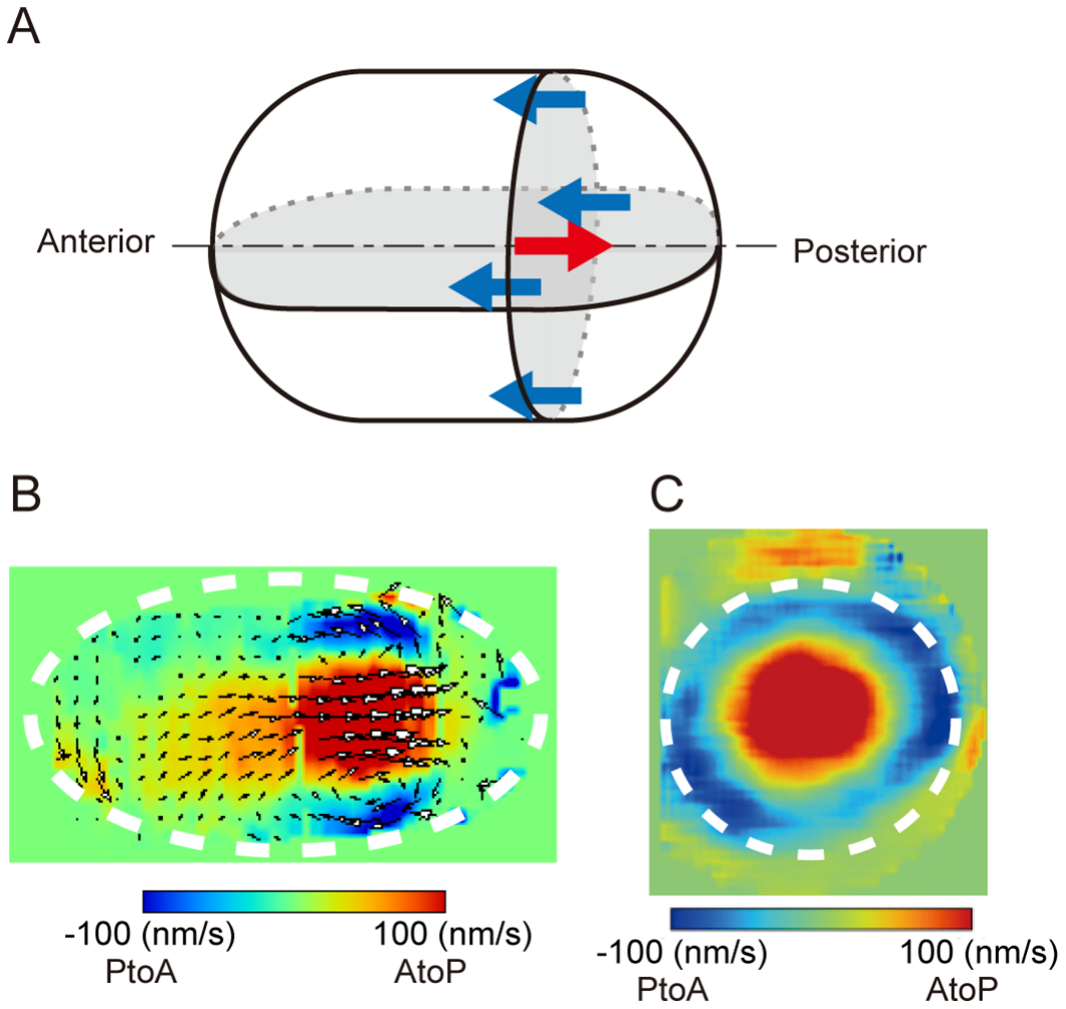
Axial symmetry of cytoplasmic streaming in *C*. *elegans* embryos. (A) Scheme of 3D flow in *C*. *elegans* embryos. Flow direction near the cell cortex and at the center are shown in blue and red arrows, respectively. (B, C) Flow field on a plane parallel (B) or perpendicular (C) to the AP axis; the field was quantified by PIV analysis carried out using SPIM images. White dotted ellipses approximately indicate the borders of the embryo.

In this study, we assumed that cytoplasmic streaming is axially symmetric, which has been demonstrated in mice [7]. To demonstrate that cytoplasmic streaming is also axially symmetric in *C*. *elegans*, we performed fast 3D image acquisition by MuVi-SPIM [19] (S3 Movie), which allowed us to quantify for the first time the entire 3D flow velocity during cytoplasmic streaming (Fig 3). We quantified velocity distribution in 3D using custom-written 3D PIV software; the velocity field thus determined was consistent with previously reported dynamics of NMY-2-dependent flow [8,11]. We detected bidirectional motion of the cytoplasm in which velocity was directed anteriorly in the cortical and posteriorly in the cytoplasmic region (Fig 3A and 3B). Moreover, the maximal flow speed was ~0.1 μm/s; the speed was higher in the posterior than in the anterior half of the cell. To determine whether the speed of cortical flow varies depending on the angle relative to the AP axis, we quantified the flow velocity distribution in a plane perpendicular to the source-drain axis; the speed of flow was found to be distributed concentrically (Fig 3C), demonstrating that cytoplasmic streaming in *C*. *elegans* embryos exhibits axial symmetry.

### 3D hydrodynamic simulation of cytoplasmic streaming based on a streaming function-vorticity method

We solved the Stokes’ equation using the stream function-vorticity method [21], which allowed us to calculate the velocity field of 3D but axially symmetric cytoplasmic flow driven by a defined magnitude of shear stress at the cortex. This method is suitable for simulating streaming given that cytoplasmic streaming in *C*. *elegans* embryos (Fig 3) and mouse oocytes [7] is axially symmetric. For the purposes of the present study, the streaming function-vorticity method was more advantageous than was the 3D particle-based method used for simulating cytoplasmic streaming in our previous study [8], not least because the computational time required for one round of simulation was 10–100 times shorter.

### DA procedure for estimating cortical shear stress driving cytoplasmic streaming: a benchmark test

We integrated the simulation based on the streaming function-vorticity method into a DA procedure in order to estimate the distribution of cortical shear stress in vivo from experimentally derived data for the cytoplasmic streaming flow field (Fig 2B). The DA procedure estimated the shear-stress distribution by fitting the simulation results to data in the framework of Bayesian statistics [16] by performing hydrodynamic simulations using the shear-stress distribution of ~120,000 patterns sampled from a probability distribution function that was set a priori. Posterior probability values of each shear-stress distribution—which quantify the similarity between simulated and in vivo-quantified total cytoplasmic velocity fields—were then calculated. Finally, we calculated the estimated shear-stress distribution as a weighted average of the top 100 shear-stress patterns with the highest posterior probability values.

We performed a benchmark test on our DA procedure in order to test whether it could be used to estimate a shear-stress distribution whose resultant flow velocity field was known. When a non-dimensional shear-stress distribution defined as *τ*_0_(*z*) = 3.0 × (1 − *z*^2^)^0.5^ was applied to the cortex of a unit sphere with radius 1.0, a stream with a cortical velocity of *u*_0_(*z*) = (1 − *z*^2^)^0.5^ was generated [23]. Here, *z* specified the position along the source-drain axis and the positions of the source and drain poles were *z* = +1 and −1, respectively (Fig 1C). For the benchmark test, input velocity-field data was generated by calculating velocity field under the shear-stress distribution *τ*_0_(*z*), and then we asked if our method could infer *τ*_0_(*z*) from the input. The results of the test showed that the estimated stress distribution was almost identical to *τ*_0_(*z*) (Fig 2C), providing evidence for the reliability of our estimation method.

### Estimation of shear-stress distribution driving cytoplasmic streaming in *C*. *elegans* embryos

**Fig 4:**
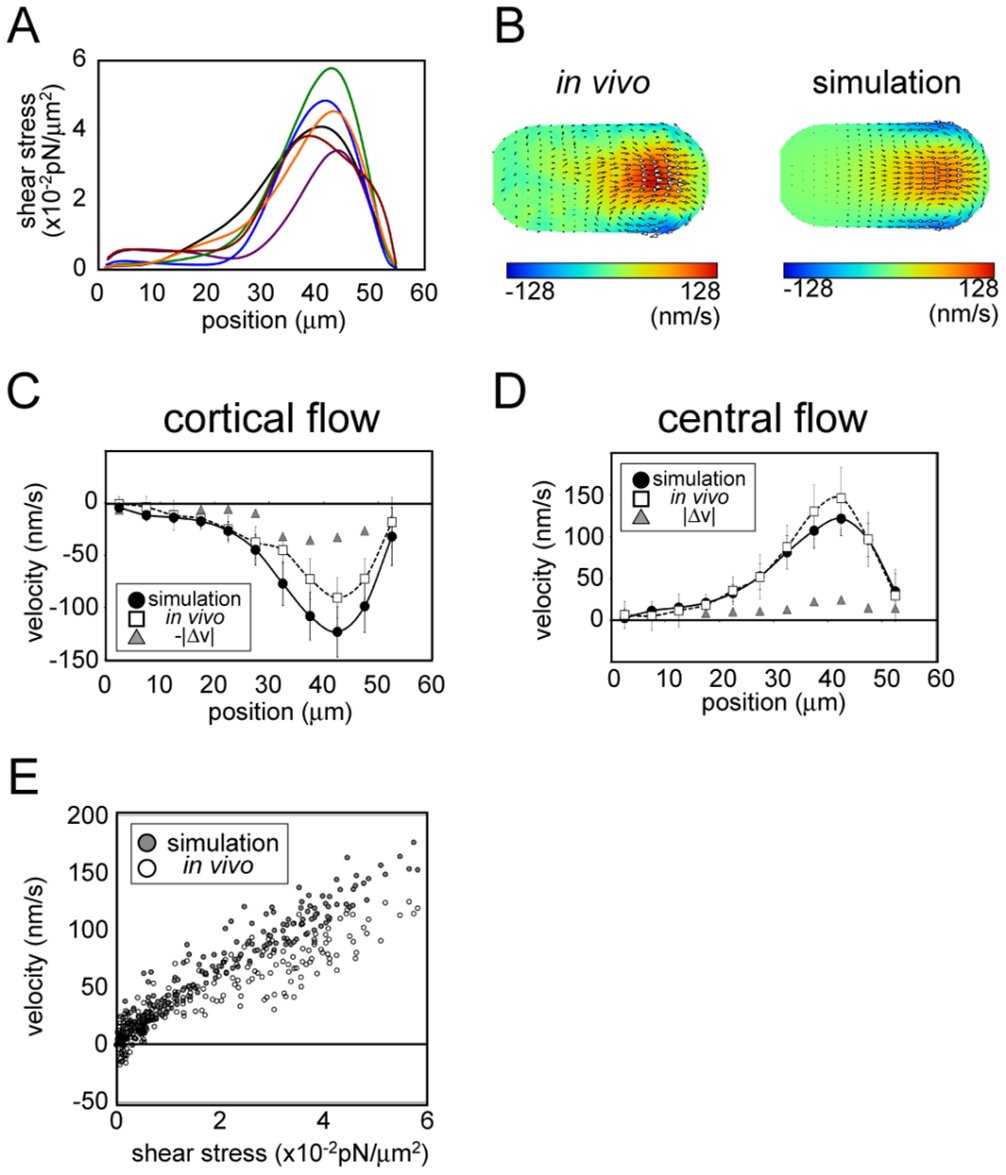
Estimation of shear-stress distribution in *C*. *elegans* embryos. (A) The shear-stress distribution estimated based on the proposed method. The fitting was performed for six embryos (indicated using distinct colors). The horizontal axis shows the position along the drain (anterior)-source (posterior) axis, with 0 indicating the drain pole. (B) Color map of the flow field measured experimentally for an embryo (left) and that of the simulation performed using the shear-stress distribution estimated using data from the same embryo. The map was normalized relative to maximal velocity. (C, D) Velocity distribution along the central source-drain axis (C) and cell cortex (D) in vivo and in the simulation performed using estimated shear stress. Velocity component and position were projected onto the middle AP axis. In vivo and simulation data represent average values from six embryos and the corresponding six fitted simulations, respectively. Error bars represent one standard deviation (simulation: black; in vivo: gray). Velocities values are positive or negative when directed towards the source and drain poles, respectively. Average differences between in vivo and simulation velocities are indicated by gray triangles. (E) Velocity along the cell cortex in the simulation and in vivo at individual positions along the cell surface, plotted against estimated shear stress at the same position.

We used our DA procedure to estimate the shear-stress distribution driving cytoplasmic streaming 5 to 7 min before pronuclear meeting in *C*. *elegans* embryos (Fig 4A) [8]. Velocity fields in the entire cytoplasm—which was the input of the stress inference—were quantified using the 2D PIV method as described in our previous study [8].

We calculated the absolute magnitude of shear stress based on our estimation results and the following physical parameters of the *C*. *elegans* one-cell stage embryo: flow velocity, ~100 nm/s; cell size, ~30 μm along short axis; and cytoplasmic viscosity, ~10 Poise [24]. The results indicated that the maximum amplitude of shear stress was in the order of 0.01 pN/μm^2^. It should be noted that the absolute value of shear stress depends on the accuracy of cytoplasmic viscosity, and must be revised when a precise measurement of cytoplasmic viscosity is obtained [25].

We calculated the velocity field generated by the estimated stress distribution based on our hydrodynamic simulation. The likelihood of the simulation results using the estimated parameters was higher than that using shear stress proportional to cortical velocity (S1 Table). This confirmed that our method improved the fit of the simulation to the experimental data as compared to simple conjecture that flow velocity and shear stress are proportional at a given location on the cell surface. Fig 4B shows the flow field in a representative embryo and in the simulation using the estimated shear stress for the same embryo. We compared the velocity along the source-drain axis measured in vivo and that in the simulation determined based on estimated shear stress (Fig 4C and 4D) and found an overall similarity between the flow fields obtained from the simulation and the *in vivo* flow fields. The result indicated that the shear-stress distribution in vivo was well estimated. However, there was also a slight difference between the flow fields; cortical flow was slower whereas cytoplasmic flow was faster in vivo than in the simulation. This may be due to unknown mechanisms enabling shear stress in vivo to be transmitted more efficiently towards the cytoplasmic interior.

A plot of the estimated shear stress and flow velocity at various locations on the cell surface indicated that a largely proportional relationship existed between the two parameters in the *C*. *elegans* embryo, except in the low-stress region (shear stress < 0.5 × 10^−2^ pN/m^2^) (Fig 4E), where the velocities were slightly higher than those expected based on the high-stress region. This can be explained by a hydrodynamic interaction whereby flow in one region is propagated to another region. Therefore, shear stress and velocity are not exactly proportional to each other.

### Estimation of shear-stress distribution driving cytoplasmic streaming in mouse oocytes

**Fig 5:**
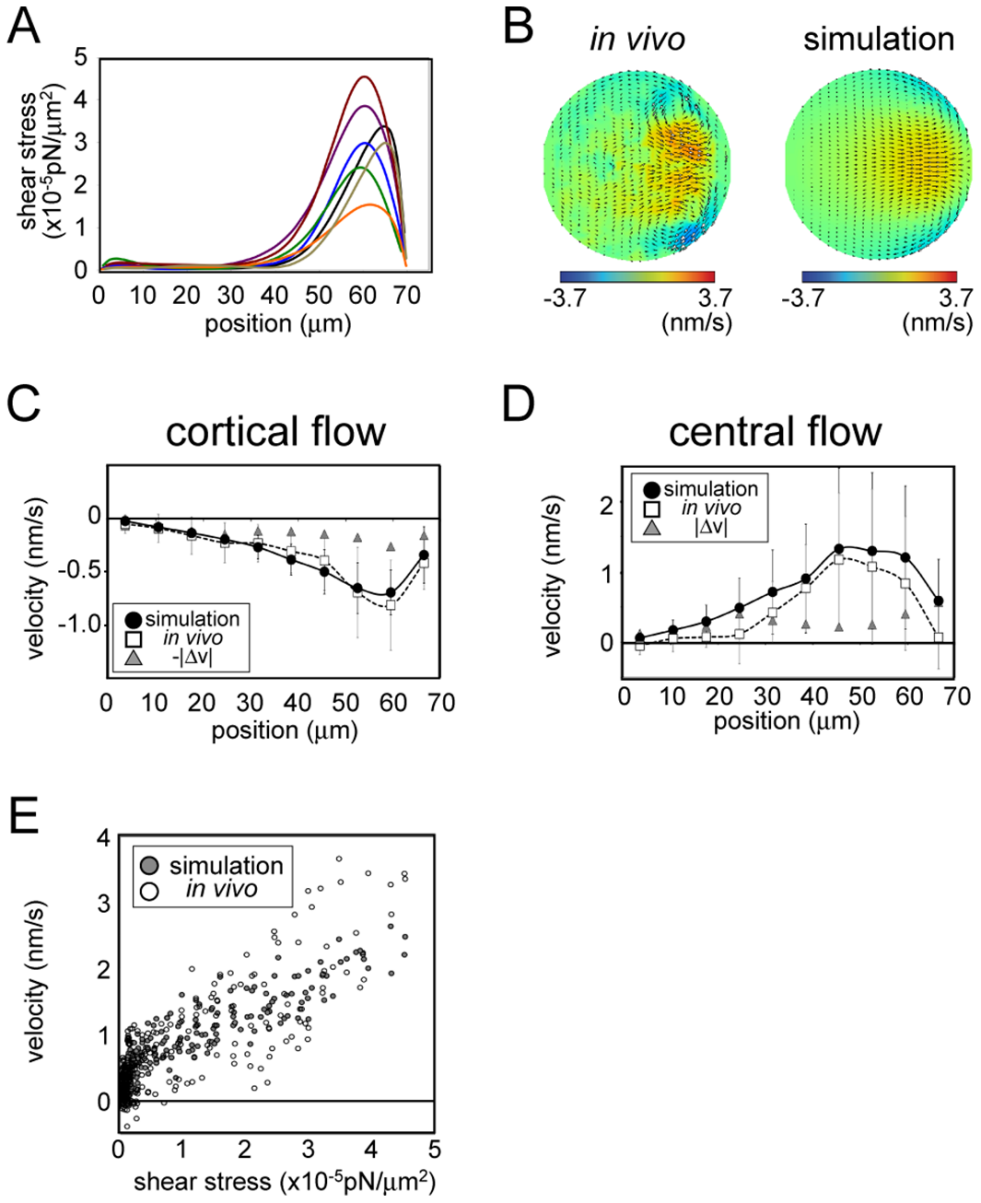
Estimation of shear-stress distribution in mouse oocytes. Estimated shear-stress distribution (A), velocity distribution (B–D), and stress-velocity relationship for cytoplasmic streaming in mouse oocytes are shown as in Fig 4 for streaming in *C*. *elegans*. The fitting was performed for seven mouse oocytes. The horizontal axis in (B–D) shows the position along the drain-source (actin cap) axis, with 0 indicating the drain pole.

To test whether our method is applicable to cytoplasmic streaming in other species, we estimated the shear-stress distribution causing streaming in mouse oocytes during meiosis II. A previous study described a hydrodynamic simulation of this process [7]. However, the velocity flow field quantified in vivo has not yet been systematically fitted to the simulation results, and shear-stress distribution has not been estimated. The fitting was achieved by first quantifying the flow velocity distribution of cytoplasmic streaming in oocytes based on phase-contrast microscopy images of the equatorial section (S2 Movie) using our 2D PIV program (Fig 5B, left panel). Since the position of the meiotic spindle was considered as being near the source pole [7], we used image sequences of oocytes whose meiotic spindles were located near the equatorial section. We assumed the 3D flow field to be axially symmetric based on a previous study [7]. The measured speed was high near the source pole, as previously reported [7]. The flow velocity of cytoplasmic streaming was two orders of magnitude slower in mouse oocytes than in *C*. *elegans*. Importantly, we detected a steady cortical flow in a region within ~50 μm of the drain pole (Fig 5C). This flow distribution did not coincide with the strong localization of actin and Arp2/3 in a region at least ~50 μm away from the drain pole [7].

We used our DA procedure to estimate the shear-stress distribution on the cell surface of seven mouse oocytes (Fig 5A). The estimated shear stress were found to be three orders of magnitude smaller than those of the *C*. *elegans* embryo, reflecting the slower flow speed (~1 nm/s) and smaller viscosity (1 Poise) of mouse oocyte cytoplasm [26]. The velocity distribution of the stream throughout the cell was closely recapitulated in the simulation that was based on estimated shear stress (Fig 5B–5D). This concurrence suggests that shear stress at the cell cortex is the primary force driving cytoplasmic streaming in mouse oocytes. A plot of estimated shear stress as a function of resultant cortical flow velocity showed a convex curve (Fig 5E); the nonlinearity was more pronounced than that observed in *C*. *elegans* embryos (Fig 4E).

The shear stress estimated by fitting using DA was concentrated near the source pole (at least ~50 μm away from the drain pole; Fig 5A). This was in contrast to the gradual change in velocity observed along the source-drain axis (Fig 5C and 5D), but was consistent with the localization of actin and Arp2/3 in vivo—i.e., at least ~50 μm away from the drain pole [7]. The agreement between the estimated shear-stress distribution and localization of active force generators supports the notion that our method can locate active forces in actual biological systems. Our analysis provides evidence that active forces generated at the actin cap on the cell cortex is transmitted via hydrodynamic properties of the cytoplasm, driving cell-wide streaming in the mouse oocyte during meiosis II.

### Relative decreases in pressure near the source pole are greater in mouse oocytes than in *C*. *elegans* embryos

**Fig 6:**
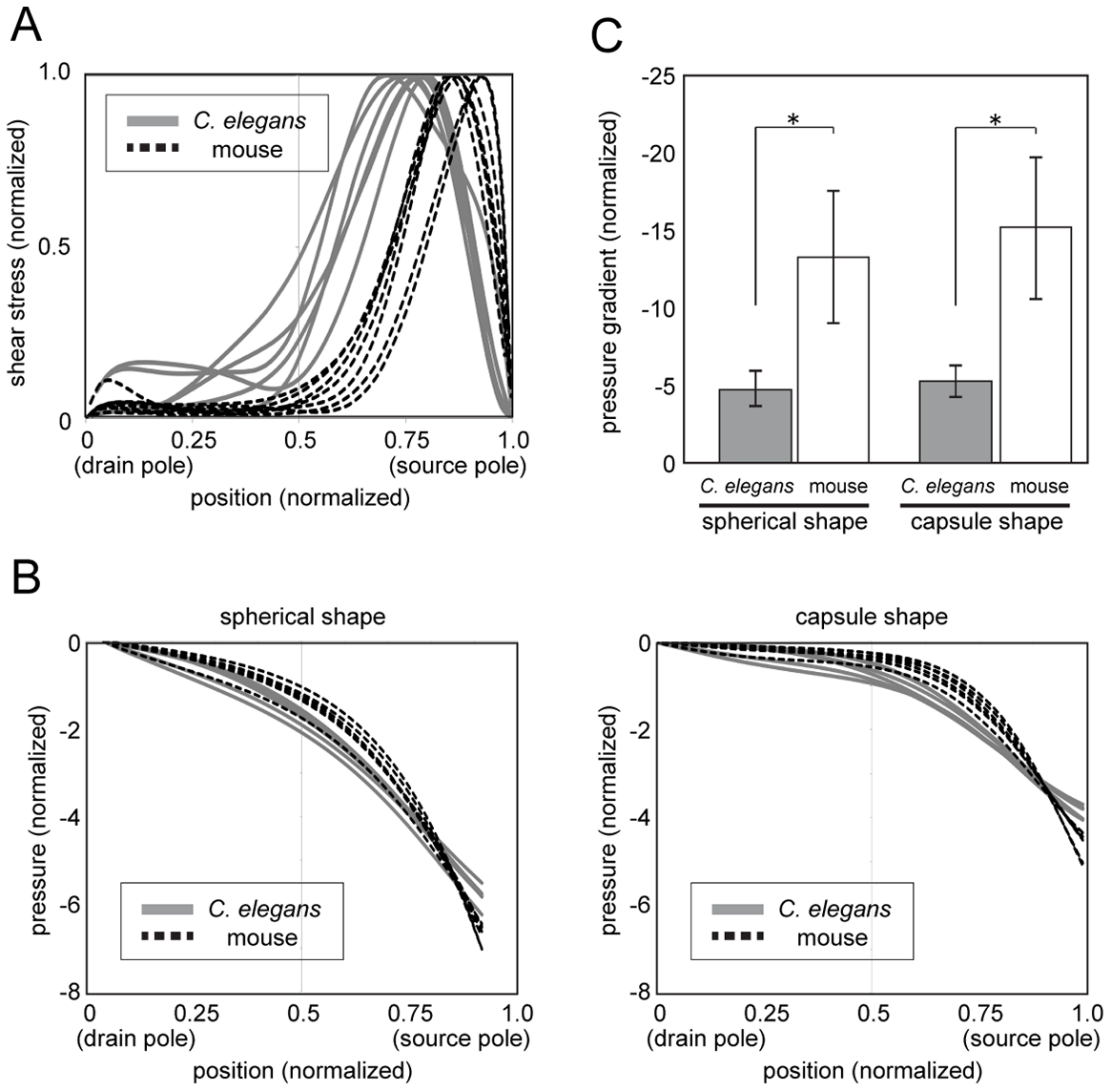
Shear-stress distribution in mouse oocytes contributes to the generation of a pressure gradient that enables the positioning of the meiosis II spindle near the cell surface. (A) Comparison of shear-stress distributions of the *C*. *elegans* embryo and mouse oocyte showing that shear stress is localized closer to the cell periphery in the latter. (B) Pressure when flow is generated using the estimated shear stress plotted against source-drain position. The plot shows that the gradient is steeper when we assume the shear-stress distribution in the mouse oocyte rather than that in the *C*. *elegans* embryo in both spherical- and capsule-shaped cells. (C) Comparison of the pressure gradient at the source end in Fig 6B; the pressure gradient is steeper in the mouse oocyte than in the *C*. *elegans* embryo. *P < 0.005 (t test, assuming non-equal variance).

The normalized shear-stress distributions in mouse oocytes and *C*. *elegans* embryos revealed that shear stress was concentrated to a greater degree at the source pole in the former than in the latter (Fig 6A). We therefore examined whether the pole-proximal shear-stress localization observed in mouse oocytes helps to maintain the localization of the meiotic spindle at the cell periphery, which is a previously proposed role of cytoplasmic streaming in mouse oocytes [7]. We performed the hydrodynamic simulation inside either a spherical- or capsule-shaped cell where the total amount of shear stress in the cell was normalized but shear-stress distributions were proportional to those estimated in *C*. *elegans* embryos (Fig 4A) and mouse oocytes (Fig 5A). We found the pressure gradient was steeper in the latter than in the former (Fig 6B and 6C). Given that this gradient is considered as a major force responsible for maintaining the spindle position proximal to the source pole [7], the localized pressure gradient in the mouse oocyte may ensure effective spindle positioning, which is a feature that may have been acquired during the course of evolution.

## Discussion

Differences in estimates of the spatial distribution of shear stress driving cytoplasmic streaming in mouse and *C*. *elegans* (Fig 6A) may reflect the distinct functions of this process in the two species. In mouse oocytes, cytoplasmic streaming is critical for maintaining the meiotic spindle near the cortical actin cap (source pole); the focused shear-stress distribution near the source pole and corresponding pressure gradient (Fig 6) are favorable for this function. In contrast, shear stress is more broadly distributed in the *C*. *elegans* embryo, which may be preferable for the establishment of cell polarity, which is the proposed function of streaming in *C*. *elegans* [12]. The difference in shear-stress ranges measured in the two species can be explained by the localization of force generators responsible for streaming. In *C*. *elegans*, streaming is thought to be driven by the contractile force of myosin, which is initially localized across the entire cell cortex [5]. Conversely, in mouse oocytes, the size of the actin cap harboring the Arp2/3 complex is considerably smaller than the uncovered area [7].

Our estimation revealed that hydrodynamic interactions play a critical role in determining flow speed, as driving forces and flow velocity at cortical regions were not proportional (Fig 5E). The nonlinear relationship can be explained by a hydrodynamic interaction. In mouse oocytes, the cortical flow velocity peaked at a position ~60 μm from the drain pole and declined gradually towards the pole (Fig 5C). In contrast, shear stress peaked at ~60 μm and decreased sharply as it approached the drain pole, reaching almost background level at a position ~40 μm from the pole (Fig 5A). The actin cap containing Arp2/3 proteins that drive streaming localize to a limited region near the source pole [7], which coincides with the position where shear stress was localized according to our estimate. Thus, active force generation that is limited to the actin cap is sufficient for generating a hydrodynamic flow with a wider range.

Our estimation method can be used to estimate cellular forces within just a few hours; such a rapid calculation will allow us to handle large amounts of data on cytoplasmic streaming and can contribute to our understanding of cell mechanics. For example, our model can be applied to the analysis of streaming in *C*. *elegans* in which specific genes have been knocked down, which can provide information on their role in the generation of active forces during cytoplasmic streaming. Recently, tissue-level cytoplasmic flow beyond the cell boundary was reported during ventral-furrow formation in *Drosophila* [27]. This flow exhibited a fountain-like flow field and can be explained based on laminar flow hydrodynamics, which is similar to the cytoplasmic streaming characterized in this study. Thus, the method presented here can be applied to the estimate of force generated by apical constriction during fly gastrulation.

## Conclusion

We established a computational method for inferring the localization of active force generators based on live-imaging data of the flow field in cytoplasmic streaming. Applying the method to cytoplasmic streaming in the *C*. *elegans* embryo and mouse oocyte revealed that cortical force generation and flow velocity at a given location were not always proportional. In mouse oocytes, shear stress induced in a small region of the cell cortex—which coincided with the actin cap—were sufficient to drive the cell-wide cytoplasmic streaming. Hydrodynamic flow is involved in many biological processes; our method can serve as a powerful tool for estimating the position and magnitude of active force generation driving these flows. In particular, correlating local shear-stress amplitude with local dynamics of active force generators such as actin can provide insight into the mechanics of hydrodynamic flow at the molecular level.

## Supporting Information

S1 Code PIV for 3D data. A code and its manual are included in the zip file.
(ZIP)

S2 Code PIV for 2D data. A code and its manual are included in the zip file.
(ZIP)

S3 Code Inference of shear stress using the DA method. Six codes and their manuals are included in the zip file.
(ZIP)

S1 Dataset Experimental data of flow fields in cytoplasmic streaming in *C*. *elegans* embryos and mouse oocytes. The dataset was used as input for the inference in this study. The dataset is in Excel format and contains 13 sheets: six (Ce1 to Ce6) are from six individual *C*. *elegans* and seven (Mm1 to Mm7) are from seven individual mice. Each sheet consists of four columns: *D(Sc(z))*, *Sc(r)*, *v*_*z*_, and *v*_*r*_, where *z* and *r* are coordinates of the position along and perpendicular to the drain-source axis, respectively. *v*_*z*_ and *v*_*r*_ are *z*- and *r*-axis components of the flow velocity, respectively. *Sc*(*z*) and *Sc*(*r*) are the scaled *z* and *r*, respectively, obtained by multiplying scaling factors (as described in “Materials and Methods”). *D*(*Sc*(*z*)) is the distance between the point *Sc*(*z*) and the source pole in the *z*-direction.
(XLSX)

S1 Fig B-spline interpolation and prior distribution. (PDF)

S1 Movie Cytoplasmic streaming in *C*. *elegans* embryos. Raw microscopic images of cytoplasmic streaming in *C*. *elegans* embryos, in which yolk granules were visualized using VIT-2::GFP.
(MOV)

S2 Movie Cytoplasmic streaming in mouse oocytes. Raw microscopic images of cytoplasmic streaming in mouse oocytes (see *Visualization of cytoplasmic streaming in mouse oocytes* in “Materials and Methods”).
(MOV)

S3 Movie 3D visualization of cytoplasmic streaming in *C*. *elegans* embryos. VIT-2::GFP. *C*. *elegans* embryos were visualized in utero with MuVi-SPIM. Scale bar = 10 μm.
(AVI)

S1 Table Improvement of log-likelihood of parameters by the estimation method. (DOC)
