# Supplementary material for: Bayesian Inference of Forces Causing Cytoplasmic Streaming in Caenorhabditis elegans Embryos and Mouse Oocytes
Source: PLoS One. 2016 Jul 29;11(7):e0159917. doi: 10.1371/journal.pone.0159917 (PMC4966953; doi:10.1371/journal.pone.0159917)
Supplement: S1 Code — A code and its manual are included in the zip file. (ZIP) [file pone.0159917.s001.zip › S1_Code_PIV_3D/README_S1.pdf]

S1 Code. PIV for 3D data

The PIV_multi_mpi.cc program can quantify the velocity field in a 3D image sequence

Environment	• A PC cluster with a Linux OS is required.<br>• MPI must be installed in the PC cluster.<br>• This program includes tiffio.h from libtiff library; therefore, libtiff library must be installed.
Input	• Images must be in .tif format.<br>• Image sequences must be divided according to time.<br>• All input files must be kept in a common folder.
Output	• Output files will be named "130321_3DPIV_WORM02_EMB04_LEFT00_param3_cytoxxxxT0yyyy.txt, in which the velocity from t = xxxx to t = yyyy is output.<br>• The first to fourth columns of the output files are x-, y-, and z-axis components of the velocity.<br>• The prefix "130321_3DPIV_WORM02_EMB04_LEFT00_param3_cyto", can be modified on line 230.
Parameters	• Line 16: kansoku is the distance between two mutually adjacent points in the x and y directions.<br>• Line 17: kensa is (xy side length of the interrogation window – 1)/2. This value is larger than 14.<br>• Line 18: kensa_z is (z side length of the interrogation window – 1)/2<br>• Line 19: kouho is the range of the search for motion in the xy direction.<br>• Line 20: kouho_z is the range of the search for motion in the z direction.<br>• Line 24: def_tate must be set as the height of the image.<br>• Line 25: def_yoko must be set as the width of the image.<br>• Line 26: def_takasa is the number of slices in the image.<br>• Line 27: sentou_file_num is the number allocated to the initial image. For example, to process numbered images from image_100.tif to image_200.tif, sentou_file_num = 100.<br>• Line 84: dirname[MAX_CHAR_NUM] must be set as the name of the folder in which data is stored.
Usage	1. Data must be stored in one folder and PIV_multi_mpi.cc in another folder.<br>2. Set all parameters.<br>3. Compile PIV_multi_mpi.cc as an MPI source code.<br>4. Run the execution file from the command line.
