# Supplementary material for: Bayesian Inference of Forces Causing Cytoplasmic Streaming in Caenorhabditis elegans Embryos and Mouse Oocytes
Source: PLoS One. 2016 Jul 29;11(7):e0159917. doi: 10.1371/journal.pone.0159917 (PMC4966953; doi:10.1371/journal.pone.0159917)
Supplement: S2 Code — A code and its manual are included in the zip file. (ZIP) [file pone.0159917.s002.zip › S2_Code_PIV_2D/README_S2.pdf]

S2 Code. PIV for 2D data

The PIV_130122.cc program can quantify the velocity field that appears in a 2D image sequence.

Environment	• Linux or Mac OS is needed in which the command line can be used.<br>• This program includes tiffio.h from the libtiff library, which must be installed.
Input	• Images must be in .tif format.<br>• Image sequences should be split by time.<br>• Images should be named "imagexxx_plane.tif", where xxx is a three-digit number starting from 001.<br>• All input image must be stored in a common folder.
Output	• The output files are named "PIV_130202_C3_L1_xxx.txt.<br>• The first, second, third, and fourth columns of the output files are x- and y-axis components of the velocity. The origin of the coordinate is the lower-left vertex of the image.<br>• The prefix "PIV_130202_C3_L1_" can be modified on line 119.
Parameters	• Line 19: kansoku is the distance between two mutually adjacent points where velocity is calculated.<br>• Line 20: kensa is (xy side length of the interrogation window – 1)/2. This value must be larger than 18.<br>• Line 21: kouho is the range of the search for the motion. This value must be slightly larger than the maximum displacement of objects in an image per frame.
Usage	1. Place PIV_130122.cc in the folder where images are stored.<br>2. Set all parameters.<br>3. Compile PIV_130122.cc.<br>4. Run the execution file from the command line.
