# Supplementary material for: Bayesian Inference of Forces Causing Cytoplasmic Streaming in Caenorhabditis elegans Embryos and Mouse Oocytes
Source: PLoS One. 2016 Jul 29;11(7):e0159917. doi: 10.1371/journal.pone.0159917 (PMC4966953; doi:10.1371/journal.pone.0159917)
Supplement: S3 Code — Six codes and their manuals are included in the zip file. (ZIP) [file pone.0159917.s003.zip › S3_Codes_DA/README_S3.pdf]

S3 Code. Inference of shear stress using data assimilation

Users can either link eleg_sabun.h, SFMT.c, sabun_120609_2.cc, and eleg_PF_util_SABUN_test.cc, or link eleg_sabun.h, SFMT.c, sabun_130204ap_2.cc, and eleg_PF_util_SABUN_test_130204.cc to infer shear stress either in the *C. elegans* embryo or in mouse oocyte, respectively. SFMT.c is distributed at http://www.math.sci.hiroshima-u.ac.jp/~m-mat/MT/SFMT/.

Environment	• A PC cluster with a Linux OS is required.<br>• MPI must be installed on the PC cluster.
Input	• The original PIV data is (z, r, v_z, v_r), where the origin is the drain pole. From this, generate (*Sc (z)*, *Sc (r)*, v_z, v_r)—where *Sc (z)* and *Sc (r)* are the scaled z and r, respectively—by multiplying the scaling factors as described in the Materials and Methods. The input for this software is calculated from this as (*D(Sc(z))*, *Sc(r)*, v_z, v_r), where *D(z)* is the distance between point z and the source pole in the z-direction.
Output	• FORCE2_130106.dat on line 486 in sabun_120609_2.cc (elegans), or FORCEM2_130213_6.dat at the line 494 in sabun_130204ap_2.cc records (nz_i, τ_i, i), where τ_i is the estimated shear stress, estimated shear stress distribution (z, $\tau(z)$), and (nz_i, τ_i, relative posterior, Node ID, i, log of posterior) for all 100 individual trials with the highest posterior values. Node ID was sorted in descending order of the posterior value.<br>• FORCE_130106.dat on line 434 in sabun_120609_2.cc, or FORCEM_130213_6.dat on line 442 in sabun_130204ap_2.cc records (z, $\tau(z)$), which is the average of all the trials based on posterior value.<br>• test_assim_130106.dat at the line 185 in sabun_120609_2.cc, or test_assim_130213_6.dat at the line 185 in sabun_130204ap_2.cc records (nz_i, τ_i, i) averaged by posterior*prior, prior, and posterior, respectively. These averages are of all trials. The following lines output (nz_i, τ_i, i, relative posterior*anterior, relative anterior, relativeposterior, Node ID, i, log anterior, log posterior, log posterior*anterior).<br>• Output file names can be modified.

Parameters	In eleg_sabun.h<br>• Line 20: neet must be same as the number of nodes used in the cluster.<br>• Line 15: je must be 14 or 13 for the *C. elegans* embryo and mouse oocyte, respectively.<br>• Line 16: ie must be 56 or 25 for the *C. elegans* embryo and mouse oocyte, respectively.<br>• Line 26: A_scale must be 0.000013 for the *C. elegans* embryo or 1.0 for the mouse oocyte.<br>• Line 27: U_scale must be a value close to the maximal speed in the PIV data<br>• Line 32: max_rep must be 936. (max_rep * neet) is the total number of the calculation. In our estimation (max_rep, neet) = (936, 128).<br>• Line 33: halflength = 55/26 or 1.0 for the *C. elegans* embryo and mouse oocyte, respectively.<br><br>In sabun_120609_2.cc<br>• Line 188: the path to the PIV data must be provided.<br>• Line 305–311: value of the average τ_i (i = 1~7) of the prior distribution must be provided.<br><br>In sabun_130204ap_2.cc<br>• Line 189: path to the PIV data must be provided.<br>• Line 313–319: value of the average τ_i (i = 1~7) of the prior distribution must be provided.<br><br>In eleg_PF_util_SABUN_test.cc<br>• Line 116: the standard deviation of τ_i (i = 1~7) must be provided.<br><br>In eleg_PF_util_SABUN_test_130204.cc<br>• Line 116: the standard deviation of τ_i (i = 1~7) must be provided.
Usage	1. Place the data and programs in a folder that is accessible to the PC cluster. Place SFMT.c and required header files in the folder.<br>2. Set all parameters.<br>3. To infer the force in the *C. elegans* embryo, write a Makefile that links eleg_sabun.h, SFMT.c, sabun_120609_2.cc, and eleg_PF_util_SABUN_test.cc. To infer the force in the mouse oocyte, write a Makefile that links eleg_sabun.h, SFMT.c, sabun_130204ap_2.cc, and eleg_PF_util_SABUN_test_130204.cc.<br>4. Using the Makefile, compile the files above as an MPI source code.<br>5. Run the execution file from the command line.
