# Supplementary material for: Bayesian Inference of Forces Causing Cytoplasmic Streaming in Caenorhabditis elegans Embryos and Mouse Oocytes
Source: PLoS One. 2016 Jul 29;11(7):e0159917. doi: 10.1371/journal.pone.0159917 (PMC4966953; doi:10.1371/journal.pone.0159917)
Supplement: S1 Fig — (PDF) [file pone.0159917.s005.pdf]

S1 Figure. B-spline interpolation and prior distribution

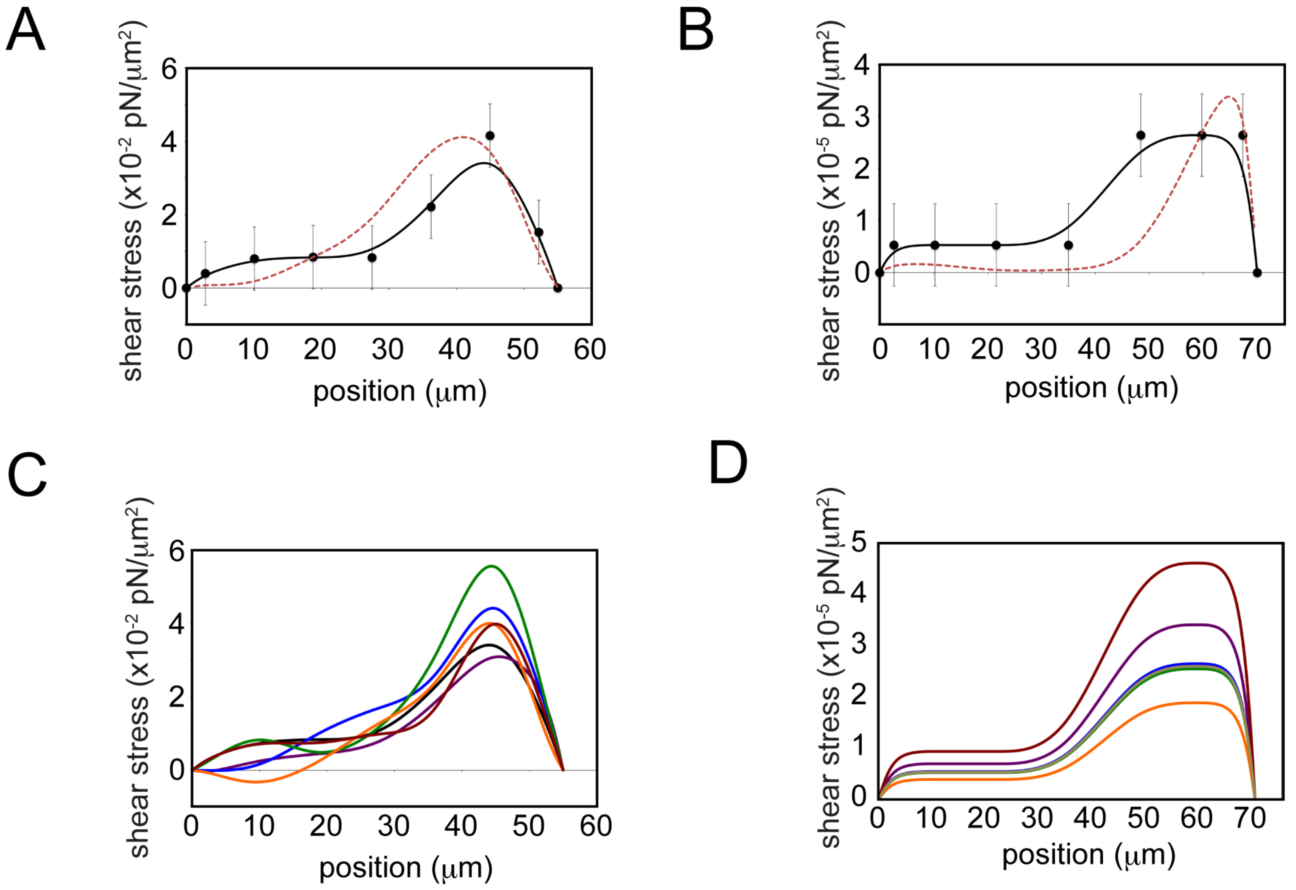

(A, B) Examples of nodes (black dots) and resultant B-spline functions (black curves) (see *Using B-spline to specify shear stress distribution* in “Materials and Methods”). Black dots and error bars indicate average and SD calculated for a Gaussian distribution of nodes used as the prior distribution with *C. elegans* (A) and mouse (B) data (see *Setting prior values* in “Materials and Methods”). Red dotted curves show the estimated optimum distribution. (C, D) Prior distributions obtained for different samples are shown in distinct colors for *C. elegans* (C) and mouse (D). Curves are B-spline functions calculated from the average node positions of prior distributions [black curves in (A) and (B)].
