# Supplementary material for: Bayesian Inference of Forces Causing Cytoplasmic Streaming in Caenorhabditis elegans Embryos and Mouse Oocytes
Source: PLoS One. 2016 Jul 29;11(7):e0159917. doi: 10.1371/journal.pone.0159917 (PMC4966953; doi:10.1371/journal.pone.0159917)
Supplement: S1 Table — (DOC) [file pone.0159917.s009.doc]

**S1 Table.** Improvement of log-likelihood of parameters by the estimation method

|  | ID | improvement | Estimated shear stress | Shear stress proportional to cortical velocity |
| --- | --- | --- | --- | --- |
| *C. elegans* | 1 | +58 | 970 | 913 |
|  | 2 | +36 | 998 | 962 |
|  | 3 | +17 | 643 | 626 |
|  | 4 | +56 | 1,163 | 1,107 |
|  | 5 | +9 | 1,001 | 991 |
|  | 6 | +21 | 679 | 658 |
| Mouse | 1 | +151 | 1,700 | 1,549 |
|  | 2 | +88 | 2,146 | 2,058 |
|  | 3 | +288 | 2,267 | 1,979 |
|  | 4 | +45 | 1,793 | 1,749 |
|  | 5 | +87 | 2,415 | 2,328 |
|  | 6 | +224 | 2,048 | 1,824 |
|  | 7 | +45 | 1,418 | 1,373 |
